# 22 Monitoring of juvenile idiopathic arthritis: don’t miss the foot deformities!

**DOI:** 10.1093/rheumatology/keac496.018

**Published:** 2022-10-07

**Authors:** L Kharrat, H Ferjani, M Moalla, W Triki, D Ben Nessib, K Maatallah, D Kaffel, W Hamdi

**Affiliations:** 1 Rheumatology Department, Kassab Orthopedics Institute, Ksar Saïd, Tunisia; 2 Faculty of Medicine, University Tunis el Manar, Tunisia

## Abstract

**Background:**

Foot deformities seem to be frequent in children with Juvenile Idiopathic Arthritis (JIA) [1,2]. These deformities can deeply affect the child’s activity and alter his quality of life.

**Objectives:**

To study the association between disease activity and foot deformities in JIA patients.

**Methods:**

We conducted a cross-sectional study including patients meeting the International League of Associations for Rheumatology (ILAR) 2001 criteria for JIA. For each patient, we collected the following data: age, disease duration, tender joint count (TJC), swollen joint count (SJC), Patient Global Assessment (PGA), Visual Analogic Scale (VAS), and therapeutic management. Disease activity was assessed using the Juvenile Arthritis Disease Activity score (JADAS). Foot deformities were assessed using an optical podoscope. C-reactive protein (CRP) and Erythrocyte sedimentation rate (ERS) levels were measured. Statistical analysis was performed using SPSS software.

**Results:**

We included 35 patients. Forty-three percent of the patients were boys (*n* = 15). The mean age was 12.2 ± 3.61 years. The mean disease duration was 4.1 ± 3.29 years. The mean PGA and the mean VAS were 3.4 ± 3.02 and 3.37 ± 2.92, respectively. The mean TJC and the mean SJC were 1.48 ± 1.69 and 0.61 ± 0.77, respectively. The mean CRP and ESR were 7.51 ± 11.85 mg/l and 18.88 ± 15.53 mm, respectively. Twenty-four patients were under non-steroidal anti-inflammatory drugs (69%), 12 patients were under methotrexate (34%), and 5 patients were under TNFα inhibitor (14%).

The mean JADAS was 7.58 ± 6.3. Seventeen percent of the patients had the inactive disease (*n* = 6). Foot deformities were found in 80% of the patients (*n* = 28). They were flatfoot in 40% (*n* = 14) and pes cavus in 46% (*n* = 16). These deformities were bilateral in 18 cases (51%). Hallux valgus was present in 14% of the cases (*n* = 5). Foot deformities were associated to a higher PGA (4.04 ± 3.01 *vs* 0.86 ± 1.2, *p*< 10^–3^), VAS (3.93 ± 2.94 *vs* 1.14 ± 1.46, *p* = 0.022), CRP level (8.84 ± 13.1 *vs* 2.79 ± 2.5 mg/l, *p* = 0.039), and higher JADAS (9.12 ± 6.25 *vs* 2.08 ± 1.93, *p*< 10^–3^).

**Conclusion:**

Our study showed that foot deformities are common in JIA. Interestingly, these deformities are associated with a higher CRP level and a higher disease activity. These results suggest that an early screening of foot deformities is advisable in patients with active disease.

**References**

1. Truckenbrodt H, Häfner R, von Altenbockum C. Functional joint analysis of the foot in juvenile chronic arthritis. Clin Exp Rheumatol. 1994; 12 Suppl 10: S91-96.

2. Gschwend N, Ivosevic-Radovanovic D. [The child’s foot in juvenile polyarthritis (cP)]. Orthopade. 1986; 15(3):212–9.

